# The complete mitochondrial genome of *Asparagus officinalis* L

**DOI:** 10.1080/23802359.2020.1780986

**Published:** 2020-07-02

**Authors:** Wentao Sheng

**Affiliations:** Department of Biological Technology, Nanchang Normal University, Nanchang, Jiangxi, China

**Keywords:** Mitochondrial genome, *Asparagus officinalis* L, phylogenetic tree analysis, neighbor-joining method

## Abstract

*Asparagus officinalis* L. is a horticultural plant in nature and belongs to the genus *Asparagus* of Asparagaceae family. The complete mitochondrial (mt) genome of *A. officinalis* was 492,062 bp in length with a 45.9% GC content. A total of 60 genes were annotated including 37 protein-coding genes, 17 tRNA genes, and 6 rRNA genes. Phylogenetic tree analysis using the neighbor-joining method demonstrated that *A. officinalis* was most closely related to *Allium cepa* and separated from *Beta vulgaris* subsp. *Vulgaris* based on the complete mt genome sequence.

As a horticulture plant with economical and cultural importance, a high-quality reference mitochondrial(mt) genome is currently lacking in *Asparagus officinalis* L. (Li et al. 2019). Phylogenetic analyses of mt DNA genome will be important for reconstructing the systematic classification relationship and genetic diversification studies of this genus. In this study, we sequenced the complete mt genome of *Asparagus officinalis* L. using a hybrid data of Illumina4000 and PacBio RSII platform, and the mt genome was recovered after *de novo* assembly and annotation. One gram of tender root from five individuals was sampled from the planting base of Jiangxi Academy of Agricultural Sciences((E115°27′, N28°09′), using the modified CTAB method (Li et al. 2013). Total DNA was constructed for the genomic library, then was sequenced and assembled into non-redundant contigs with SPAdes3.6.1 (Bankevich et al. 2012) and SOAPdenovo2 (Luo et al. 2012). Each contig of the mt genome was screened by Blast run, and was assembled by using Sequencher 5.4.6 (Altschul et al. 1997). Primer walking and PacBio sequencing were used to fill the gaps. Mt genome was annotated by using the Dual Organellar GenomeAnnotator (DOGMA) (Wyman et al. 2004). The complete mt genome sequence of *A. officinalis* was 492,062 bp (NCBI accession number: MT483944), with a typical circle structure and a GC content of 45.9%. The genome contains 37 protein-coding genes, including ones for NADH dehydrogenase (*nad1*, *2*, *3*, *4*, *4 L*, *5*, *6*, *7*, *9*), cytochrome c oxidase (*cox1*, *2*, *3*), cytochrome c biogenesis (*ccmB*, *C*, *Fn*, *Fc*), apocytochrome b (*cob*), ATP synthase (*atp1*, *4*, *8*, *9*), ribosomal proteins (*rpl2*, *5*, *10* and *rps1*, *3*, *4*, *7*, *10*, *12*, *13*, *19*), succinate dehydrogenase (*sdh3*, *4*), maturase and membrane transporter (*mttB* and *matR*). Meanwhile, the genome contains 17 tRNA genes and 3 rRNA genes (*rrn5, 18, 26*). To classify the systematic relationship of *A. officinalis*, the neighbor-joining phylogenetic tree was constructed using the software MEGA-X based on 8 complete mt genome (https://blast.ncbi.nlm.nih.gov) (Kumar et al. 2018). The tree showed a close relationship *between A. officinalis and Allium cepa*, and it is separated from *Beta vulgaris* subsp. *Vulgaris* (Figure 1). The complete mt genome sequence of *A. officinalis* can benefit further studies on its systematic classification.

**Figure 1.**
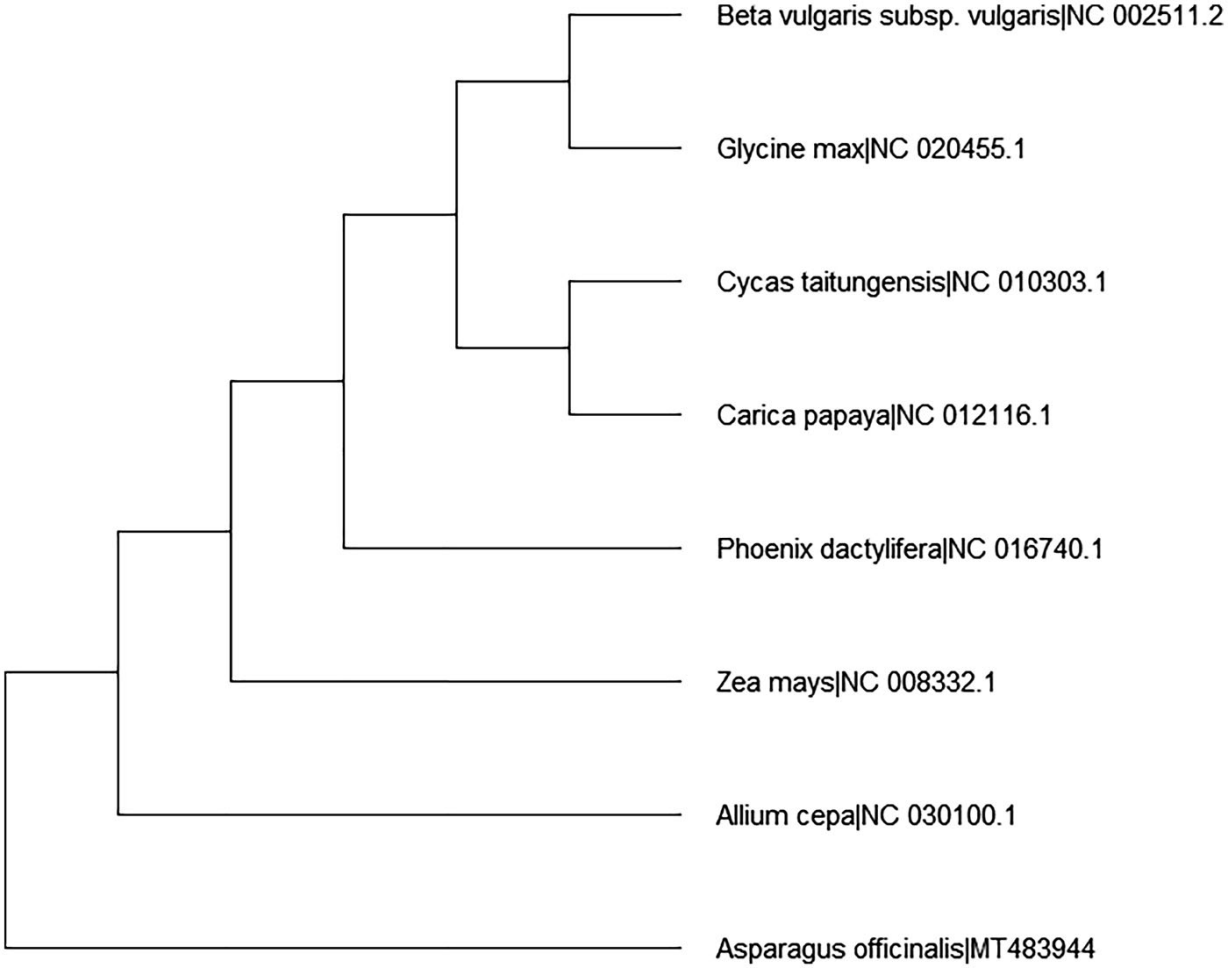
Neighbor-joining (NJ) tree based on the complete mt DNA genome sequences of 8 species.

## Data Availability

The data that support the findings of this study are openly available in [*Asparagus officinalis*] at [https://www.ncbi.nlm.nih.gov/nuccore/ MT483944], reference number [MT483944].
